# Germacrone derivatives: synthesis, biological activity, molecular docking studies and molecular dynamics simulations

**DOI:** 10.18632/oncotarget.14832

**Published:** 2017-01-27

**Authors:** Jie Wu, Yu Feng, Chao Han, Wu Huang, Zhibin Shen, Mengdie Yang, Weiqiang Chen, Lianbao Ye

**Affiliations:** ^1^ School of Pharmacy, Guangdong Pharmaceutical University, Guangzhou 510006, China; ^2^ School of Basic Courses, Guangdong Pharmaceutical University, Guangzhou 510006, China; ^3^ Inspection and Quarantine Technology Center of Zhanjiang Entry-Exit Inspection and Quarantine Bureau, Zhanjiang 524001, China; ^4^ School of Traditional Chinese Medicine, Guangdong Pharmaceutical University, Guangzhou 510006, China

**Keywords:** germacrone derivative, biological activity, c-Met kinase, molecular docking, molecular dynamics simulations

## Abstract

Germacrone is one of the major bioactive components in the *Curcuma zedoaria* oil product, which is extracted from *Curcuma zedoaria* Roscoe, known as *zedoary*. The present study designed some novel germacrone derivatives based on combination principles, synthesized these compounds, and investigated their inhibitions on Bel-7402, HepG2, A549 and HeLa cells. Meanwhile, the study evaluated inhibitions of these derivatives on c-Met kinase, which has been detected in a number of cancers. The results suggested that the majority of the compounds showed stronger inhibitory effect on cancers and c-Met kinase than germacrone. Furthermore, our docking experiments analyzed the results and explained the molecular mechanism. Molecular dynamics simulations were then applied to perform further evaluation of the binding stabilities between compounds and their receptors.

## INTRODUCTION

**Figure 1:**
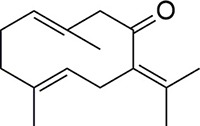
Structure of germacrone

*Rhizoma Curcuma* belongs to the *Zingiberacea* family, which is composed of about 70 species of rhizomatous herbs at home and abroad, with approximately 20 species existing in China. In China, it is traditionally used for the treatment of dyspepsia, flatulence, menstrual disorders, fever, and cough [1]. *Zedoary’s* extract have analgesic, antitumor, antimicrobial, and antiallergic activity [2–6]. Germacrone is a main bioactive constituent found in *Zedoary* oil product which is extracted from *Curcuma zedoaria* Roscoe [7]. The Germacrone (Figure 1) presents extensive bioactivities including antiulce, antiinflammatory, depressant, vasodilator, antibacterial, choleretic, antitussive, antitumor, antifeedant, antifungal and hepatoprotector effects [8–10].

The c-Met signaling pathway plays imperative roles in embryogenesis and early development; whereas c-Met is expressed by most carcinomas and its elevated expression relative to normal tissue has been detected in a number of cancers [11–13]. Activation of the HGF/c-Met signaling pathway has been shown to lead to a wide array of cellular responses including motility, survival, scattering, wound healing, invasion, angiogenesis, proliferation, tissue regeneration and branching morphogenesis [14–16]. Therefore, c-Met has become an attractive target of antitumor therapy.

**Figure 2:**
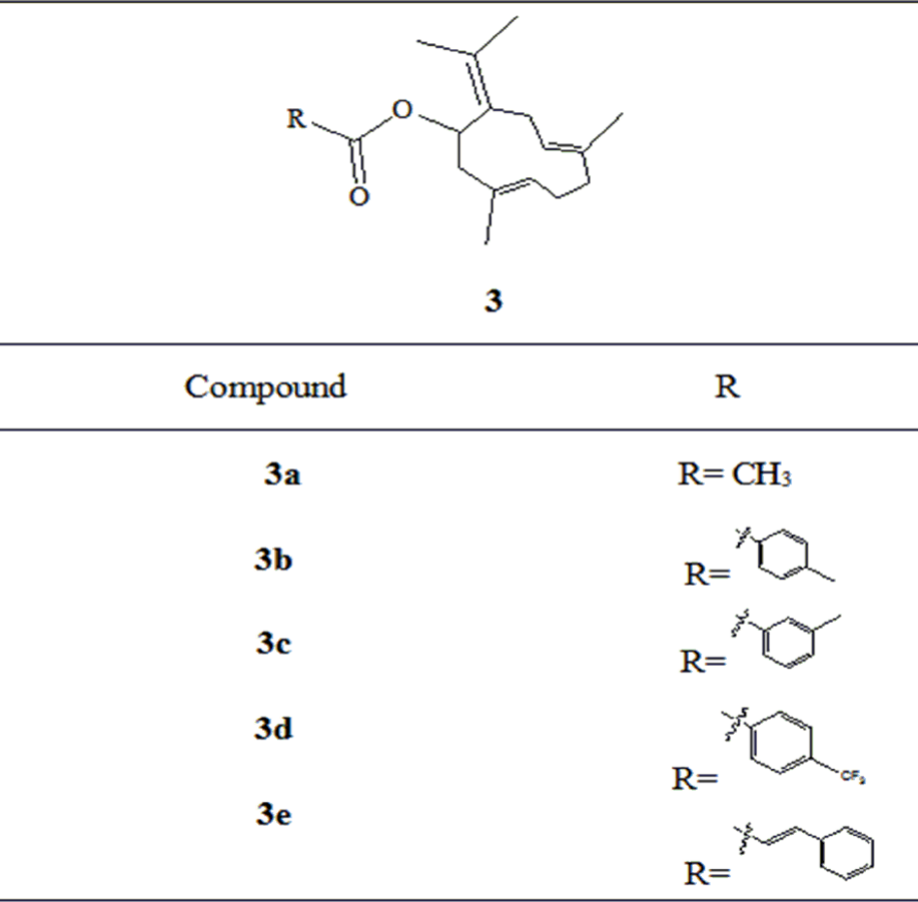
Germacrone derivatives

References reported that carboxylic esters, especially aromatic esters, had strong anticancer activity. In the present study, carboxylic esters were introduced to germacrone to obtain novel germacrone derivatives (3a–3e) (Figure 2) based on the combination principles. It is hoped that introduction of carboxylic esters can increase anticancer activity of germacrone. Hopefully, it can absorb and distribute to various tissues quickly.

In the present study, we synthesized these compounds and evaluated their inhibitions on Bel-7402, HepG2, A549 and HeLa cells. Meanwhile, the study investigated inhibition of these derivatives on c-Met kinase, which has been over-expressed in a number of cancers. Further, the molecular mechanisms of these compounds to c-Met kinase were explained by docking experiments. The further evaluation of the binding stabilities between compounds and their receptors were performed by molecular dynamics simulations.

## RESULTS AND DISCUSSION

### Chemistry

**Scheme 1:**
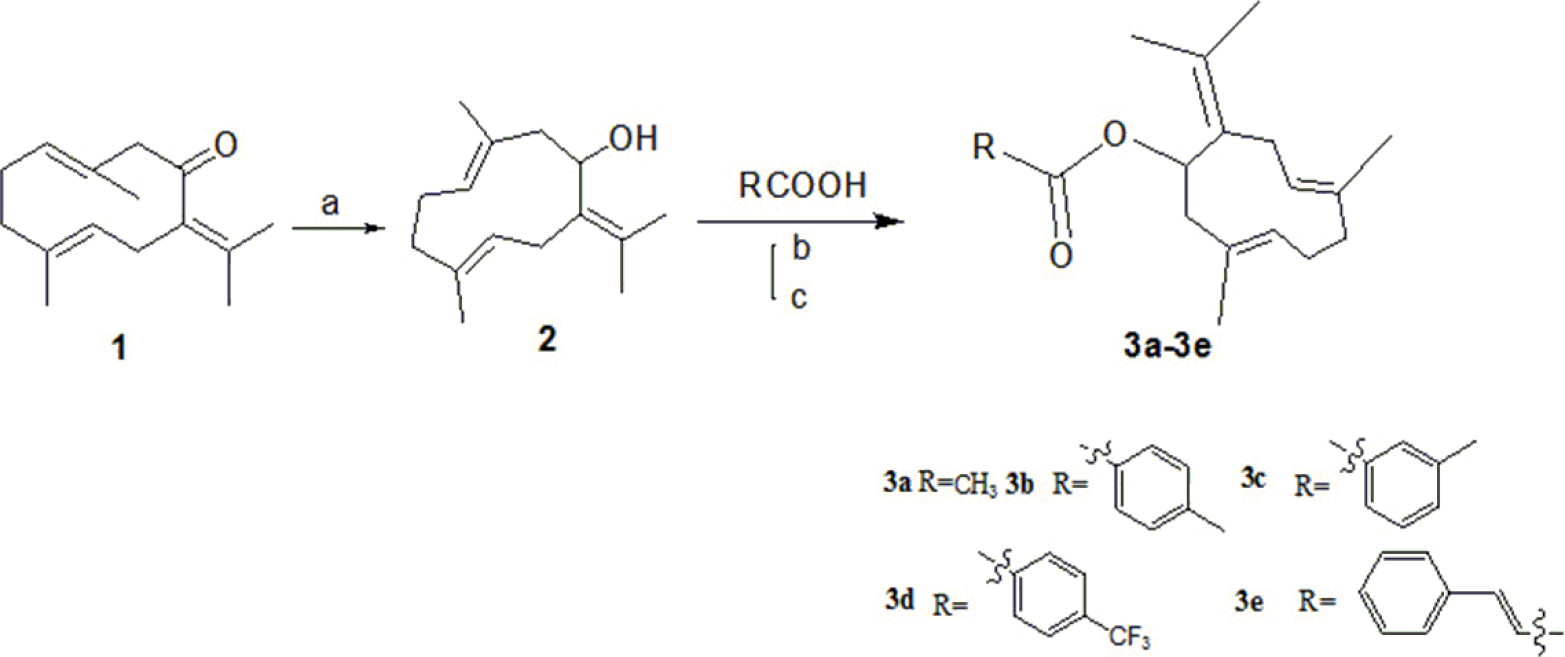
Synthesis of 3a-3e

The target compounds (2, 3a–3e) were prepared according to Scheme 1. The (*3E,7E*)-3,7-dimethyl-10-(propan-2-ylidene)cyclodeca-3,7-dien-1-yl-acetate (3a), (*3E,7E*)-3,7-dimethyl-10-(propan-2-ylidene)cyclodeca-3,7-dien-1-yl-4-methylbenzoate(3b),(*3E,7E*)-3,7-dimethyl-10-(propan-2-ylidene)cyclodeca-3,7-dien-1-yl-3-methyl- benzoate(3c),(*3E,7E*)-3,7-dimethyl-10-(propan-2-ylidene) cyclodeca-3,7-dien-1-yl-4-(trifluoromethyl) benzoate (3d), (Z)-(*3E,7E*)-3,7-dimethyl-10-(propan-2-ylidene) cyclodeca-3,7-dien-1-yl-3-phenylacrylate (3e) were obtained via DMAP/DCC esterification reaction with yields of 67%, 62%, 47%, 53%, 60% .This reaction was carried out by using dry DCM or acetonitrile as a solvent, DCC as a dehydrator and DMAP as a catalyst and can provide a range of applicability. Due to the distinct difference in polarity, these compounds can be separated easily by silica gel column chromatography. Structural modification of germacrone and a systematic study revealed that the 8-hydroxy might be further optimization studies and a range of substituent would be suitable for this position.

The derivatives were characterized by ^1^H NMR, ^13^C NMR, elemental analyses and EI-MS. The analytical data for target compounds can be seen from Experimental and ^13^C NMR and EI-MS gave information about carbon atoms and all the ion peaks corresponding to molecular weight of confirmed novel compounds.

*(3E,7E)-3,7-dimethyl-10-(propan-2-ylidene)cyclodeca-3,7-dienol* (2) Yield: 84%,^1^H NMR (400 MHz, CDCl_3_) δ 5.29 (dd, J = 11.1, 9.6 Hz, 1H), 5.2–5.1 (m, 1H), 4.4 (dd, J = 5.4, 1.9 Hz, 1H), 2.6 (dd, J = 12.6, 11.3 Hz, 1H), 2.6–2.5 (m, 1H), 2.5 (d, J = 1.9 Hz, 1H), 2.4 (m, 1H), 2.4-2.3 (m,1H), 2.32 (m,1H), 2.31-2.25 (m, 2H), 1.7 (s, 6H), 1.57 (s, 3H), 1.55 (s, 3H). ^13^C NMR(100MHz, (D_6_) DMSO) δ138.3 (C-11), 137.2 (C-4), 131.7 (C-10), 129.4 (C-1), 129.2 (C-7), 120.1 (C-5), 71.8 (C-8), 39.2 (C-3), 38.6 (C-9), 28.8 (C-6), 25.6 (C-2), 21.4 (C-12), 21.4 (C-13), 18.8 (C-14), 16.3 (C-15). EI-MS: 221.35[M+H^+^]. Anal.Calcd for C_15_H_24_O (220.18): C, 81.76; H, 10.98; O, 7.26. Found: C, 81.76; H, 10.95; O, 7.29.

*(3E,7E)-3,7-dimethyl-10-(propan-2-ylidene)cyclodeca-3,7-dien-1-yl-acetate* (3a) Yield: 67%, ^1^H NMR (400 MHz, CDCl_3_) δ 5.52 (dd, J = 5.8, 1.3 Hz, 1H), 5.31 (dd, J = 11.1, 9.6 Hz, 1H), 5.13 (dd, J = 11.3, 7.0 Hz, 1H), 2.54 (dd, J = 10.5, 6.5 Hz, 2H), 2.46 (dd, J = 15.8, 1.3 Hz, 1H), 2.39 (t, J = 4.2 Hz, 1H), 2.37 (d, J = 1.8 Hz, 1H), 2.35 (dd, J = 3.1, 1.6 Hz, 1H), 2.33 (t, J = 2.1 Hz, 1H), 2.31 (m, 1H), 2.10 (s, 3H), 1.77 (s, 6H), 1.58 (s, 3H), 1.55 (s, 3H). ^13^C NMR (100 MHz, (D_6_) DMSO) δ 169.9 (C-16), 138.3 (C-11), 137.2 (C-4), 131.7 (C-10), 129.4 (C-1), 129.2 (C-7), 120.1 (C-5), 73.7 (C-8), 39.2 (C-3), 38.6 (C-9), 28.8 (C-6), 25.6 (C-2), 21.4 (C-12, C-13), 20.9 (C-17), 18.8 (C-14), 16.3 (C-15). EI-MS: 263.20 [M+H^+^]. Anal.Calcd for C_16_H_24_O_2_ (262.19): C, 77.82; H, 9.99; O, 12.20. Found: C, 77.80; H, 9.98; O, 12.22.

*(3E,7E)-3,7-dimethyl-10-(propan-2-ylidene)cyclodeca-3,7-dien-1-yl-4-methylbenzoate* (3b) Yield: 62%, ^1^H NMR (400 MHz, CDCl_3_) δ 7.92 (dd, J = 8.5, 1.7 Hz, 2H), 7.15 (dd, J = 8.5, 1.7 Hz, 2H), 5.54 (dd, J = 5.6, 1.6 Hz, 1H), 5.32 (dd, J = 11.1, 9.6 Hz, 1H), 5.17-5.09 (m, 1H), 2.55 (d, J = 10.0 Hz, 1H), 2.52 (d, J = 8.4 Hz, 1H), 2.43 (d, J = 1.9 Hz, 1H), 2.39 (s, 1H), 2.38 (s, 1H), 2.33 (s, 1H), 2.32 (d, J = 1.6 Hz, 3H), 2.32-2.30 (m, 2H), 1.77 (s, 6H), 1.58 (s, 3H), 1.57 (s, 3H). ^13^C NMR (100 MHz, (D_6_) DMSO) δ 165.6 (C-16), 139.7 (C-20), 138.2 (C-11), 137.1 (C-4), 133.8 (C-17), 131.6 (C-10), 129.4 (C-1), 129.2 (C-7), 129.1 (C-18, C-22), 129 (C-19, C-21), 120.1 (C-5), 73.7 (C-8), 39.2 (C-3), 38.6 (C-9), 28.8 (C-6), 25.6 (C-2), 21.4 (C-12, C-13), 21.2 (C-23), 18.8 (C-14), 16.3 (C-15). EI-MS: 339.23[M+H^+^]. Anal.Calcd for C_23_H_30_O_2_ (338.22): C, 81.61; H, 8.93; O, 9.45. Found: C, 81.61; H, 8.95; O, 9.43.

*(3E,7E)-3,7-dimethyl-10-(propan-2-ylidene)cyclodeca-3,7-dien-1-yl-3-methylbenzoate* (3c) Yield: 47%,^1^H NMR (400 MHz, CDCl_3_) δ 8.00-7.97 (m, 1H), 7.96 (s, 1H), 7.50 (t, J = 8.0 Hz, 1H), 7.40-7.35 (m, 1H), 5.54 (dd, J = 5.6, 1.6 Hz, 1H), 5.32 (dd, J = 11.3, 10.0 Hz, 1H), 5.16-5.09 (m, 1H), 2.56 (dd, J = 12.3, 10.5 Hz, 1H), 2.53–2.50 (m, 1H), 2.48-2.46 (m, 1H), 2.43 (d, J = 1.7 Hz, 3H), 2.39 (s, 1H), 2.38 (d, J = 3.7 Hz, 1H), 2.32 (d, J = 1.6 Hz, 1H), 2.32-2.25 (m, 2H), 1.77 (s, 6H), 1.58 (s, 3H), 1.57 (s, 3H). ^13^C NMR (100 MHz, (D_6_) DMSO) δ 165.1 (C-16), 138.2 (C-11), 138.2 (C-19), 137.1 (C-4), 132.6 (C-22), 131.7 (C-10), 130.6 (C-18), 130.5 (C-20), 129.4 (C-1), 129.2 (C-7), 128.4 (C-21), 126.8 (C-17), 120.1 (C-5), 73.7 (C-8), 39.2 (C-3), 38.6 (C-9), 28.8 (C-6), 25.6 (C-2), 21.4 (C-12,C-13), 20.9 (C-23), 18.8 (C-14), 16.3 (C-15). EI-MS: 339.26 [M+H^+^]. Anal.Calcd for C_23_H_30_O_2_ (338.22): C, 81.61; H, 8.93; O, 9.45.Found: C, 81.62; H, 8.92; O, 9.45.

*(3E,7E)-3,7-dimethyl-10-(propan-2-ylidene)cyclodeca-3,7-dien-1-yl-4-(trifluoromethyl)benzoate* (3d) Yield: 53%, ^1^H NMR (400 MHz, CDCl_3_) δ 7.95 (d, J = 8.6 Hz, 2H), 7.86 (d, J = 8.6 Hz, 2H), 5.53 (dd, J = 5.4, 1.9 Hz, 1H), 5.32 (dd, J = 11.0, 10.0 Hz, 1H), 5.16-5.09 (m, 1H), 2.57 (dd, J = 10.4, 8.6 Hz, 1H), 2.52 (dd, J = 10.3, 7.4 Hz, 1H), 2.44-2.39 (m, 1H), 2.39 (s, 1H), 2.38-2.34 (m, 2H), 2.32 (d, J = 1.6 Hz, 1H), 2.32-2.25 (m, 1H), 1.77 (s, 6H), 1.58 (s, 3H), 1.51 (s, 3H). ^13^C NMR (100 MHz, (D_6_) DMSO) δ 165.6 (C-16), 138.3 (C-11), 137.2 (C-4), 133.8 (C-17), 132 (C-20), 131.6 (C-10), 129.4 (C-1), 129.2 (C-7), 129.1 (C-18, C-22), 125.1 (C-19, C-21), 124 (C-23), 120.1 (C-5), 73.7 (C-8), 39.2 (C-3), 38.6 (C-9), 28.8 (C-6), 25.6 (C-2), 21.4 (C-12, C-13), 18.8 (C-14), 16.3 (C-15). EI-MS: 393.20[M+H^+^]. Anal.Calcd for C_23_H_27_F_3_O_2_ (392.20): C, 70.39; H, 6.93; O, 8.15. Found: C, 70.39; H, 6.95; O, 8.15.

*(Z)-(3E,7E)-3,7-dimethyl-10-(propan-2-ylidene)cyclodeca-3,7-dien-1-yl-3-phenylacrylate* (3e) Yield: 60%, ^1^H NMR (400 MHz, CDCl_3_) δ 7.66 (d, J = 16.4 Hz, 1H), 7.45 (dd, J = 6.5, 1.6 Hz, 1H), 7.44-7.40 (m, 2H), 7.40–7.35 (m, 2H), 6.69 (d, J = 16.1 Hz, 1H), 5.46 (dd, J = 5.6, 1.6 Hz, 1H), 5.32 (dd, J = 11.1, 9.6 Hz, 1H), 5.16 – 5.09 (m, 1H), 2.56 (dd, J = 13.1, 11.3 Hz, 1H), 2.53-2.48 (m, 1H), 2.43 (d, J = 1.6 Hz, 1H), 2.40-2.38 (m, 1H), 2.38-2.34 (m, 2H), 2.33 (d, J = 1.7 Hz, 1H), 2.32-2.25 (m, 1H), 1.77 (s, 6H), 1.58 (s, 3H), 1.53 (s, 3H). ^13^C NMR (100 MHz, (D_6_) DMSO) δ 167 (C-16), 144.4 (C-18), 138.3 (C-11), 137.2 (C-4), 134.4 (C-19), 131.6 (C-10), 129.4 (C-1), 129.2 (C-7), 128.9 (C-22), 128.7 (C-21, C-23), 127.3 (C-20, C-24), 120.1 (C-5), 115.9 (C-17), 73.7 (C-8), 39.2 (C-3), 38.6 (C-9), 28.8 (C-6), 25.6 (C-2), 21.4 (C-12,C-13), 18.8 (C-14), 16.3 (C-15). EI-MS: 351.23[M+H^+^]. Anal.Calcd for C_24_H_30_O_2_ (350.22): C, 82.24; H, 8.63; O, 9.13. Found: C, 82.22; H, 8.63; O, 9.15.

### Evaluation of biological activity

**Figure 3:**
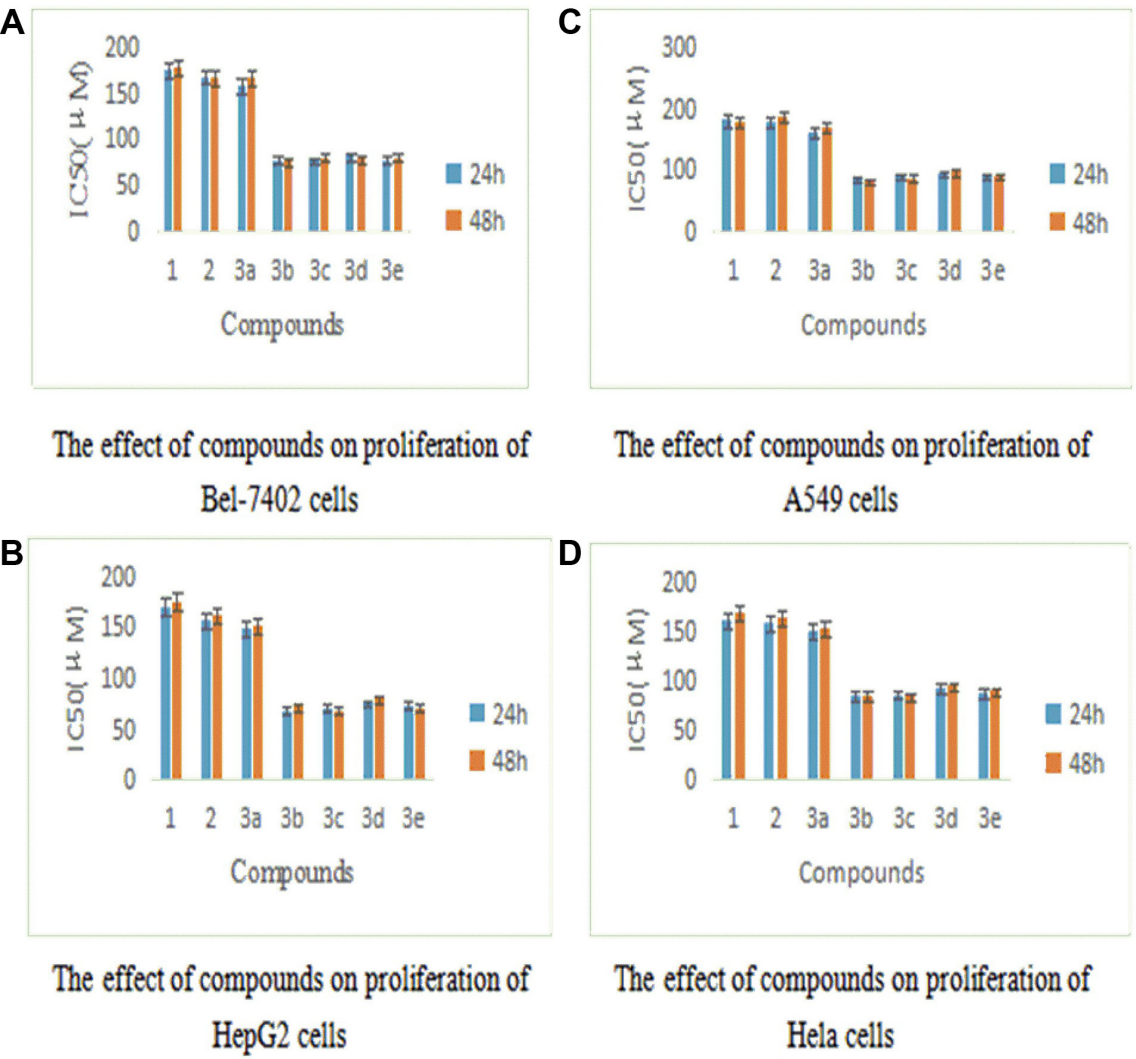
Inhibition of germacrone derivatives on Bel-7402, HepG2, A549 and Hela cells

**Figure 4:**
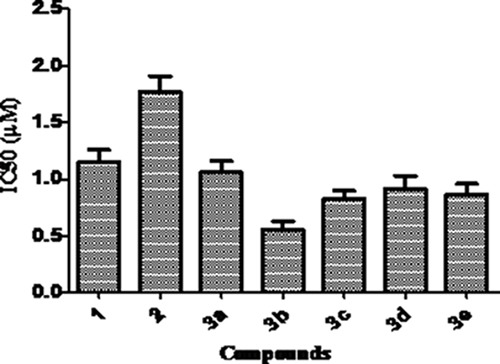
Inhibition of germacrone derivatives on c-Met kinase

Biological activities were evaluated by investigating the effect of germacrone and its derivatives on HepG2, A549, Bel-7402 and HeLa cells. Cells were treated with compounds at the concentrations of 12.5 μmol/L, 25 μmol/L, 50 μmol/L, 100 μmol/L, 200 μmol/L, 400 μmol/L and 800 μmol/L, respectively. MTT assay was then applied at 24 h, 48 h. As shown in Table 1. The proliferations of HepG2, Bel7402, A549 and HeLa cells were inhibited in a dose-dependent manner, and cytolytic activity was markedly inhibited at the same time. The growth-inhibitory effect had no significant differences after incubation with germacrone and derivatives for 24 h and 48 h (Figure 3), so there was no great influence to the anti-proliferation effect of germacrone and derivatives on Bel-7402, HepG2, A549 and HeLa cells with the extended treatment time after 24 h. However, all the compounds were active against Bel-7402, HepG2, A549 and HeLa cells to some extent. Inhibitions of germacrone derivatives (3a-3e) on these cells were stronger than germacrone and these results indicated that introduction of carboxylic esters could increase anticancer activity of germacrone, so we could elementary make sure that designed compounds should be well worth studying based on preliminary biological tests. Compound 3b was found to show highest activity against HepG2 cell line with an IC_50_ value of 68.23 μM. Since references reported that germacrone induced apoptosis by inhibiting Bcl-2 expression and inducing p53 and Bax expression and induced cell cycle arrest and apoptosis through mitochondria-mediated caspase pathway, and induced apoptosis in human hepatoma HepG2 cells through inhibition of the JAK2/STAT3 signaling pathway [17–19]. Before we began to design these compounds, we carefully studied these references. Firstly, we hoped that designed compounds had stronger broad spectrum anti-tumor activities so we investigated their inhibitions on Bel-7402, HepG2, A549 and HeLa cells compared to germacrone. The results showed that these compounds, especially compound 3b had better effect on HepG2 cell than other cells. Then we investigated mechanism according to above-mentioned documents, but the pre-experimental results showed no effect reported in these references. Hu C T, et al., Wang SY, et al. and Xie B, et al. reported that HepG2 was inhibited by inhibiting c-Met/HGF signaling pathway. Because c-Met kinase is expressed in a number of cancers and our research area is major in finding novel c-Met inhibitors so we investigated inhibition of germacrone derivatives on c-Met kinase [20–22]. The results of compounds on c-Met kinase showed that germacrone derivatives were active against c-Met kinase with IC_50_ values of 1.06 μM, 0.56 μM, 0.83 μM, 0.92 μM, 0.87 μM, respectively (shown in Table 2). In accordance with the results of cells, the IC_50_ values of 3a-3e were lower than germacrone, in which 3b had the best inhibitory effect with IC_50_ value of 0.56 μM (Figure 4, Supplementary Table 1). The results suggested that derivatives had good activity so we could initially confirm that designed compounds might well repay investigation on the basis of preliminary activity tests. Further research works on activities are currently under investigation and will be reported in due course.

**Table 1:** IC_50_ values of compounds on Bel-7402, HepG2, A549 and Hela cells

Compound	Bel-7402 IC_50_(μM)		HepG2 IC_50_ (μM)		A549 IC_50_ (μM)		Hela IC_50_ (μM)	
	24 h	48 h	24 h	48 h	24 h	48 h	24 h	48 h
**1**	173.54 ± 1.53	177.21 ± 1.97	169.52 ± 2.07	174.56 ± 1.88	179.97 ± 2.14	177.21 ± 1.87	160.69 ± 1.54	167.78 ± 1.81
**2**	167.08 ± 1.91	166.32 ± 1.83	155.2 ± 1.75	161.54 ± 1.32	177.21 ± 1.80	185.24 ± 1.99	157.31 ± 1.47	163.15 ± 1.73
**3a**	157.32 ± 1.33	166.25 ± 1.51	147.67 ± 1.47	151.59 ± 1.71	162.2 ± 1.55	168.87 ± 1.74	149.32 ± 1.33	152.95 ± 1.65
**3b**	75.85 ± 0.96	74.16 ± 0.83	68.23 ± 0.69	71.47 ± 0.82	84.85 ± 0.71	81.87 ± 0.88	83.64 ± 0.73	83.78 ± 0.76
**3c**	75.5 ± 1.05	78.55 ± 0.51	70.21 ± 0.87	67.84 ± 0.48	88.52 ± 0.53	87.21 ± 0.94	84.1 ± 0.71	82.54 ± 0.64
**3d**	80.14 ± 1.19	77.56 ± 0.77	74.66 ± 0.99	78.85 ± 0.77	93.48 ± 0.79	95.93 ± 0.97	91.25 ± 0.87	93.31 ± 0.90
**3e**	75.87 ± 0.81	79.22 ± 0.89	72.48 ± 0.64	69.98 ± 0.68	90.11 ± 0.75	88.21 ± 0.81	86.55 ± 0.80	88.24 ± 0.84

**Table 2:** IC_50_ values of compounds against c-Met kinase

Compound	1	2	3a	3b	3c	3d	3e
IC_50_ (μM)	1.15	1.77	1.06	0.56	0.83	0.92	0.87

### Docking studies

In pre-experiment, we carried out docking experiment by using several pdb proteins including 3DKF, 2WGJ, 2RFS and 3DKC. Only 3DKC interacted with germacrone and derivatives owing to sesquiterpene structures and 3DKC was used frequently in our experiment so we chose it to make docking experiment. The 3DKC is the crystal structure of c-Met kinase in complex with ATP and its information is seen from http://www.rcsb.org/pdb/explore/materialsAndMethods.do?structureId = 3DKC.

**Figure 5:**
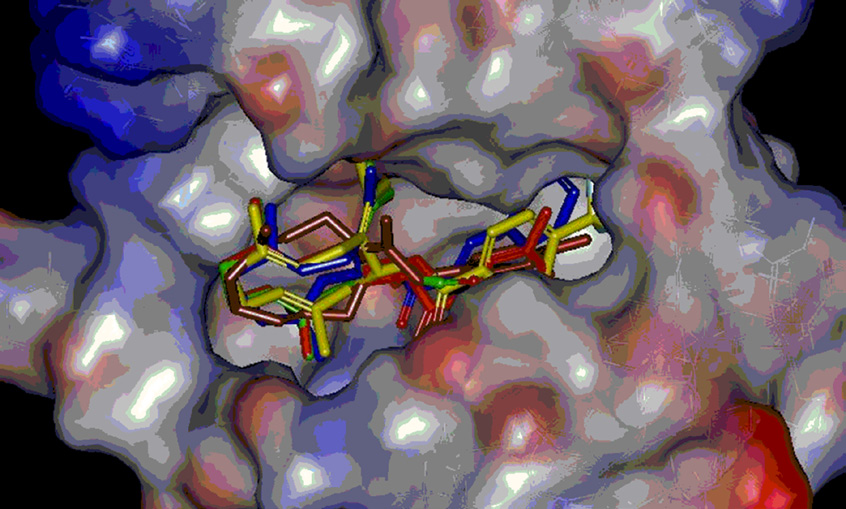
Compact binding modes of all compounds (PDB code: 3dkc)

**Figure 6:**
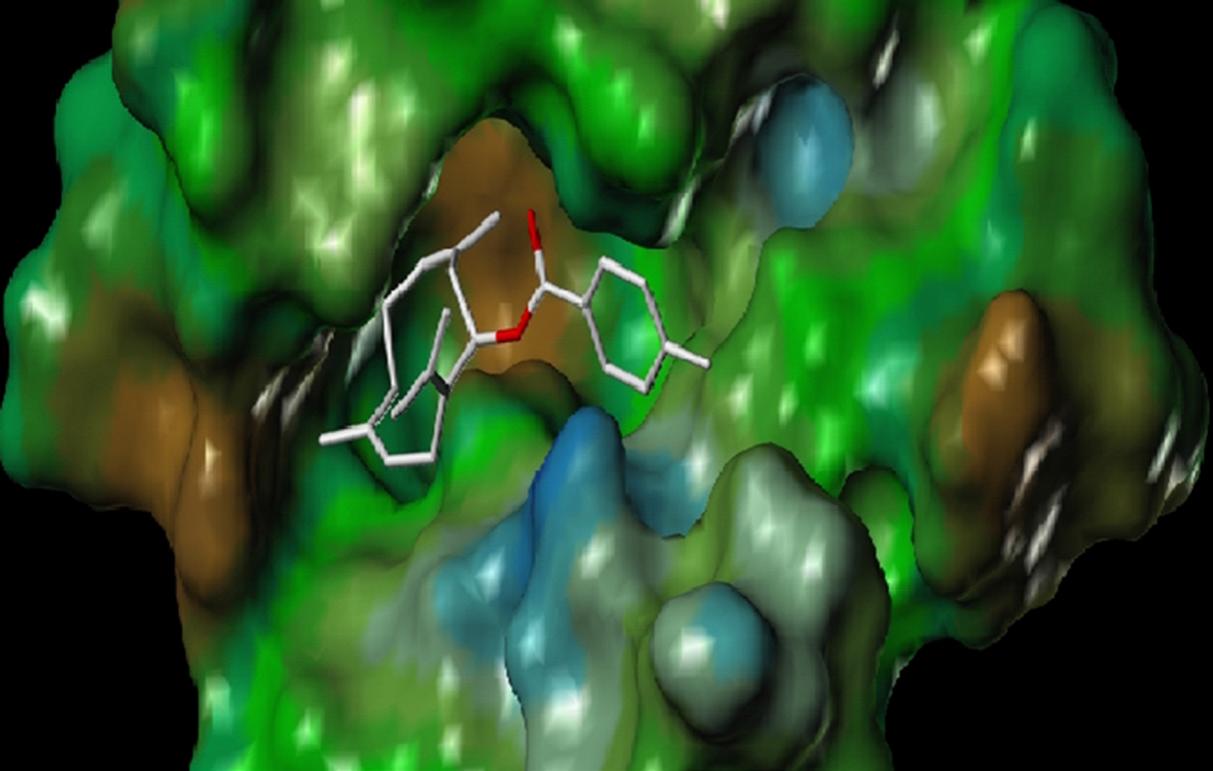
Conformation of 3b

Docking experiments indicated that there was a binding mode between our synthesized compounds and the active site of protein 3dkc (shown in Figure 5). The (E, E)-1, 5-cyclodecadiene system interacted with the protein crystal 3dkc strongly by hydrophobic force and π-π stacking interaction of the benzene ring group. Compound 3b showed highest activity owing to hydrophobic interaction of methyl as shown in Figure 6. The binding energies were shown in Table 3. The compound 3b also had stronger binding energies and inhibition, in which R was methyl substituted benzene.

**Table 3:** Binding energies of complexes between compounds and 3DKC

Compounds	R	c-Met Inhibition rate (%)	-CDOCKER energy (Kcal/mol)
**1**	-	27%	29.9587
**2**	-OH	23%	22.5548
**3a**	Me	31%	22.8715
**3b**	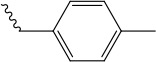	56%	65.2361
**3c**	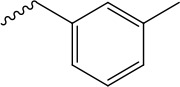	47%	51.0112
**3d**	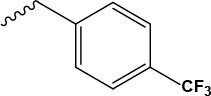	38%	40.1487
**3e**	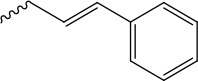	41%	45.2356

### MD simulations and ΔGpred calculation

**Figure 7:**
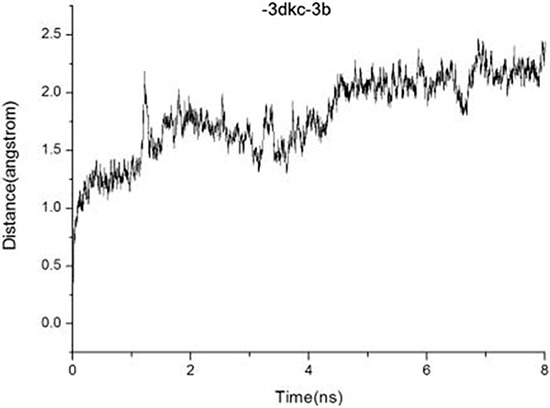
Plots of RMSD for all of the backbone atoms (Å) vs simulation time (ns) for 3DKC in complex with 3b

Molecular docking experiments gave a probably momentary binding mode that could be unreasonable and unstable. Molecular dynamics simulations were applied to perform further study of binding stabilities between compound (1-3e) and 3DKC. The crystal structures of 3DKC complex with 1-3e were used to evaluate the reliability of MD simulations. The RMSD curve and the surface area curve of the 1 ns indicated that 3b was relatively stable and the trajectories were well smoothly and other compounds were unstable with uneven trajectories (shown in Figure 7 and Supplementary Figure 1 to Supplementary Figure 7 ). The MD parameters were appropriate for the MD simulations.

The 2 3DKC-ligand complexes were performed by 8 ns MD simulations under identical MD conditions and all compounds gave stable RMSD curves during their MD simulations. ΔGpred were selected and ordered or 3DKC inhibitory activity assessment which values more negative than −25 kcal/mol. The predicted ΔGpred value of the 3b compound was −28.36 kcal/mol while predicted ΔGpred value of other compounds were more than −25 kcal/mol (shown in Table 4).

**Table 4:** The value of -CDOCKER energy

Compounds	R	c-Met Inhibition rate (%)	-DeltaGpred Energy (kcal/mol)
**1**	-	27%	16.35
**2**	-OH	23%	12.31
**3a**	Me	31%	19.69
**3b**	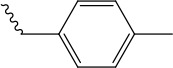	56%	28.07
**3c**	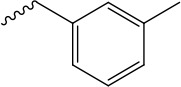	47%	27.14
**3d**	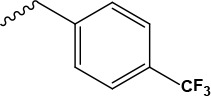	38%	20.88
**3e**	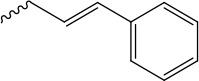	41%	21.28

## MATERIALS AND METHODS

### Chemicals and reagents

All chemicals were obtained from Aladdin or J&K. Solvents were purified and dried by standard procedures, and stored over 3-Å molecular sieves. Reactions were followed by TLC using SILG/UV 254 silica-gel plates. Flash chromatography (FC): silica gel (SiO2; 40 μm, 230-400 mesh). ^1^H NMR and ^13^C NMR Spectra: Bruker Digital NMR Spectrometer, rep. δ in ppm, J in Hz. EI-MS: Waters ZQ4000. Cells were obtained from China Center for Type Culture Collection of Wuhan University; c-Met kinase were purchased from Millipore (Billerica, MA); RPMI-1640culture medium and new-born calf serum from Gibco (GrandIsland,NY); Methyl thiazolyl tetrazolium (MTT)was purchased from Amresco (Solon, OH).

### Synthesis of compounds

### Synthesis of 2

The suspension of LiAlH_4_ (3.8 mg, 0.1 mmol) was added to a cold (−10°C) solution of 1 (21.8 mg, 0.1 mmol) in THF (15 ml) under argon and vigorous stirring. After 1 h (TLC control) the reaction was stopped by successive addition of water (2 drops), 6 N NaOH solution (2 drops) and water (4 drops). The mixture was extracted with DCM (3 × 20 ml) to give a crude product which was purified by silica gel column chromatography (hexane : t-butyl methyl ether, 70:30) to obtain 2 (18.3 mg, 84%).

### Synthesis of 3a

A mixture of acetic acid (9 mg, 0.15 mmol), SOCl_2_ (21.4 mg, 0.18 mmol), DMF 2 drops and DCM (5 ml) was refluxed for 4 h to obtain acetyl chloride. Acetyl chloride (11.8 mg, 0.15 mmol) was added portion wise to a solution of 2 (21.9 mg, 0.1 mmol), Py (15.8 mg, 0.2 mmol) in THF (15 ml) under nitrogen at 0°C. Then, the mixture was stirred for 4 h at r.t. (TLC control), the solution was filtered. The solvent was removed under reduced pressure, and the crude product was purified by column chromatography (ethyl/acetat, 80/20) to obtain the target product 3a (17.4 mg, 67%).

### Synthesis of 3b

A mixture of 2 (21.9 mg, 0.1 mmol), DMAP (6.1 mg, 0.05 mmol), para-toluic acid (13.6 mg, 0.1 mmol) and DCM (10 ml) was stirred for 30 min under nitrogen at −10°C, then, a solution of DCC (41.2 mg, 0.2 mmol) in DCM (5 ml) was added slowly and the mixture was stirred vigorously for 24 h at r.t. and filtered. The solvent was removed under reduced pressure to give a crude product which was purified by column chromatography to obtain the product 3b (20.8 mg, 62%).

### Synthesis of 3c, 3d and 3e

Compounds 3c, 3d and 3e were prepared in analogy to 3b.

### Cell culture

Bel-7402 cells, HepG2 cells (Human hepatoma cell line), A-549 and HeLa cells were investigated according to related reference [23] and the cells were cultured in RPMI 1640 and DMEM media supplemented with 10% FBS and 1%P/S (100 units/ml peni cillin and100 mg/ml streptomycin), respectively, at 37°C in a 5% CO_2_ atmosphere.

### Cell assay

Proliferation of cells was evaluated by MTT assay [10]. Cells were inoculated at 1.0 × 10^4^ cells/mL with 200 μL in each well of 96-well plate and allowed to adhere to the plates overnight. Then the cells were treated with a range of concentrations (0–800 μmol/L) of target products or 0.1% DMSO for 24, 48 h. 20 μL of MTT was added to each well and incubated at 37°C in dark for 4 h. After removal of the medium, cells were treated with 150 μL of DMSO and shaken for 15 min to completely dissolve the formazan crystals. The absorbances at 590 nm of the dissolved solutions were detected using a SpectraMAX190 microplate reader (Molecular Devices, USA).

### Inhibition of c-Met kinase

The inhibitions of c-Met kinase were investigated referred to a published literature [24]. The IC_50_ values were detected using TR-FRET. 50 nM 6 His-tagged recombinant human c-Met residues 974-end (Millipore) was cultured in medium containing 2.5 m mol/L MnCl_2_, 10 m mol/L MgCl_2_, 20 m mol/L Tris, 2 m mol/L DTT and 0.01% Tween 20 with 5 m mol/L ATP and 200 n mol/L 5FAM-KKK -SPGEYVNIGFG-NH_2_ with 25 μL at room temperature for 60 min. Compounds were made up to 100 μM solutions and were tested with 10 concentration gradients by two times dilutions. Each group was tested for three times Reactions were termined by IMAP stop solution. Plates were incubated for an overnight and analyzed by using AiphaQuest.

### Molecular docking

Molecular docking was performed according to related reference [25].The docking experiment was carried out with CDocker program which was connected with Accelrys Discovery Studio 2.5.5. The programs adapted an empirical scoring function and a patented searching engine [26–27]. Briefly, ligands were docked into the corresponding protein's binding site complied with protocol, which was generated by ligand from the crystal structure of 3DKC with random hydrogen atoms and GasteigereHückel charges but not water and ligands other parameters were default values except that the threshold was 1. The structure of receptor was minimized to 10,000 cycles using Powell method in DS 2.5.5. The geometries of all compounds were optimized by conjugate gradient method of TRIPOS. The convergence criterion was identified as 0.001 kcal/mol.

### MD simulations

The MD simulations were performed on the basis of molecular docking by using AMBER 10.0 for ligands and AMBER ff03 for protein, and using Gaussian 03 program to calculate partial atomic charges at a neutral pH, with histidines 164 and 200 protonated at δ position, and using SHAKE algorithm to restrict all the bonds given the time step of 2 fs and cutoff distance of 8 Å with long-range electrostatic interactions treated with the particle mesh Ewald (PME) method [28–30]. The heating operation was carried out from 0 to 300 K in 50 ps using Langevin dynamics at a constant volume and equilibrated for 100 ps at a constant pressure of 1 atm after four steps of minimizations, which included 2500 cycles of steepest descent minimization, followed by 2500 cycles of conjugated gradient minimization. Heavy atoms of receptor-ligand complex were restrained to 0, 10, 100, and 500 kcal/ (mol Å2) and were 10 kcal/ (mol Å2) during the heating and equilibration steps while solvent molecules were not restricted. Finally, periodic boundary conditions of 8 ns were performed for the whole system with normal pressure of 1 atm and normal temperature of 300 K in the production step.

### MM-PBSA estimation of binding free energy ΔGpred

For each system, ΔGpred values were calculated using 100 snapshots recorded from the last with 1 ns trajectory an interval of 10 ps by Molecular Mechanics Poisson-Boltzmann Surface Area method [31–34].

## CONCLUSIONS

The present study designed and synthesized novel compounds using germacrone as leading compound based on the combination principles. The results indicated that the major of compounds had moderate inhibition on cancer cells and c-Met kinase and introduction of carboxylic esters could increase anticancer activity of germacrone. These compounds can be further researched as antitumor leading structure and supply reference for developing anticancer with clinical value. The results of molecular docking and MD simulations were very important to explain molecular mechanism of eminent activities to c-Met kinase and binding stabilities between compounds and their receptors and these results can provide theoretical basis for further research c-Met inhibitors.

## SUPPLEMENTARY MATERIALS FIGURES AND TABLES



## Abbreviations

DMSO: dimethyl sulfoxide
THF: tetrahydrofuran
DCC: dicyclohexylcarbodiimide
DMF: N, N-dimethyl formamide
DMAP: 4-dimethylaminopyridine
NMR: Nuclear Magnetic Resonance
TCL: thinlayer chromato -graphy
DCM: dichloromethane
EI-MS: electron impact spectrometry
MTT: 3-(4, 5-dimethylthiazol-2-yl)-2,5 -diphenyltetrazolium bromide
