# Supplementary material for: Germacrone derivatives: synthesis, biological activity, molecular docking studies and molecular dynamics simulations
Source: Oncotarget. 2017 Jan 27;8(9):15149–58. doi: 10.18632/oncotarget.14832 (PMC5362474; doi:10.18632/oncotarget.14832)
Supplement: Supplementary file 1 [file oncotarget-08-15149-s001.pdf]

Germacrone derivatives: synthesis, biological activity, molecular docking studies and molecular dynamics simulations

Supplementary Materials

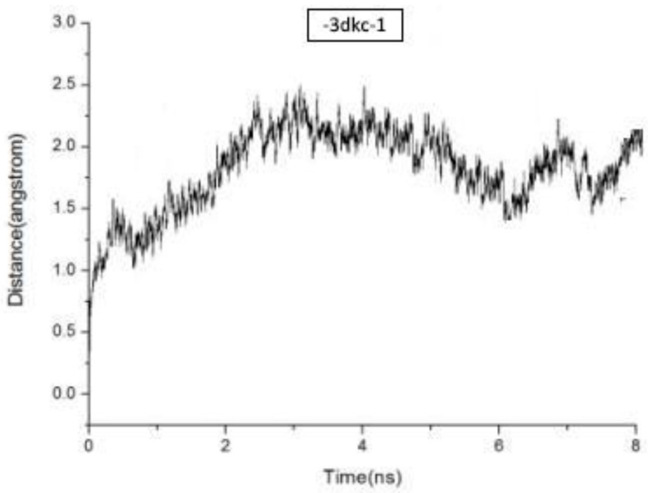

Supplementary Figure 1: Figures of Plots of RMSD for all of the backbone atoms (Å) vs simulation time (ns) for 3DKC in complex with 1.

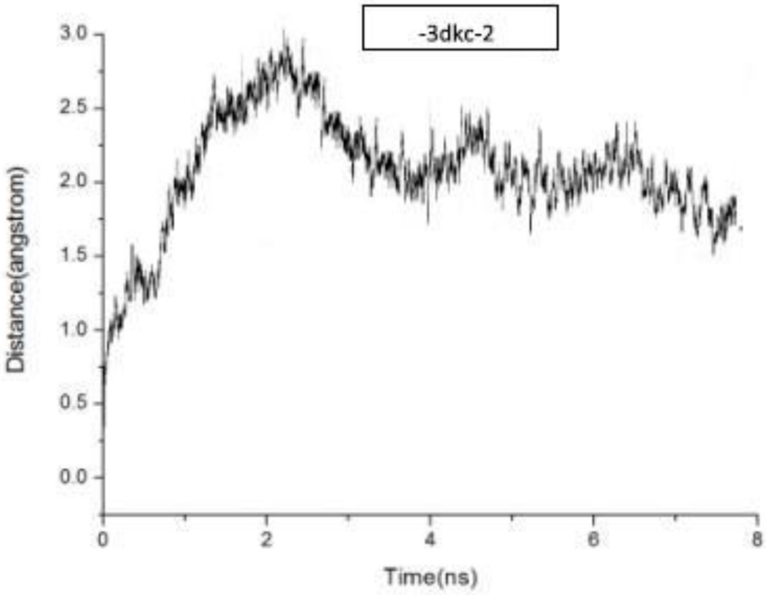

Supplementary Figure 2: Figures of Plots of RMSD for all of the backbone atoms (Å) vs simulation time (ns) for 3DKC in complex with 2.

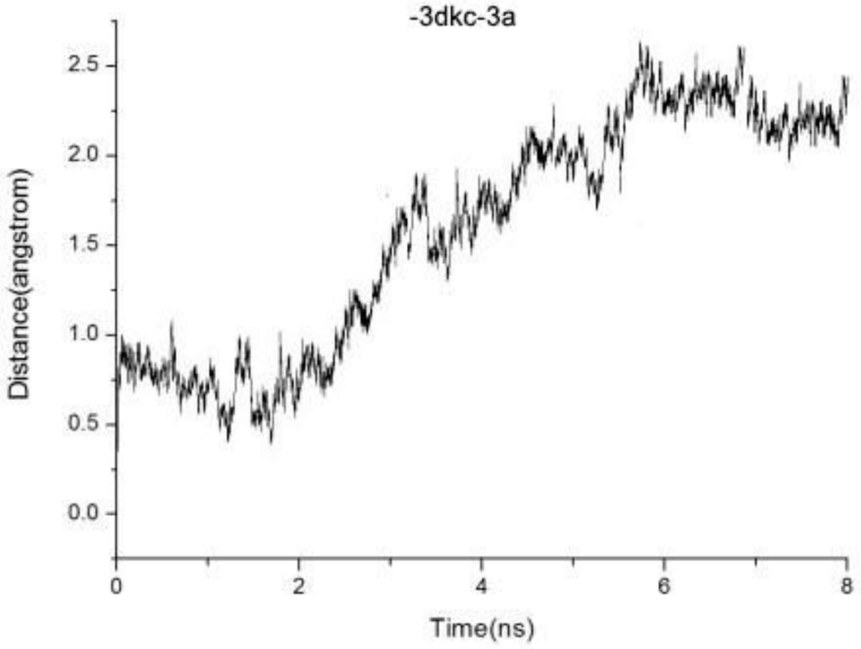

Supplementary Figure 3: Figures of Plots of RMSD for all of the backbone atoms (Å) vs simulation time (ns) for 3DKC in complex with 3a.

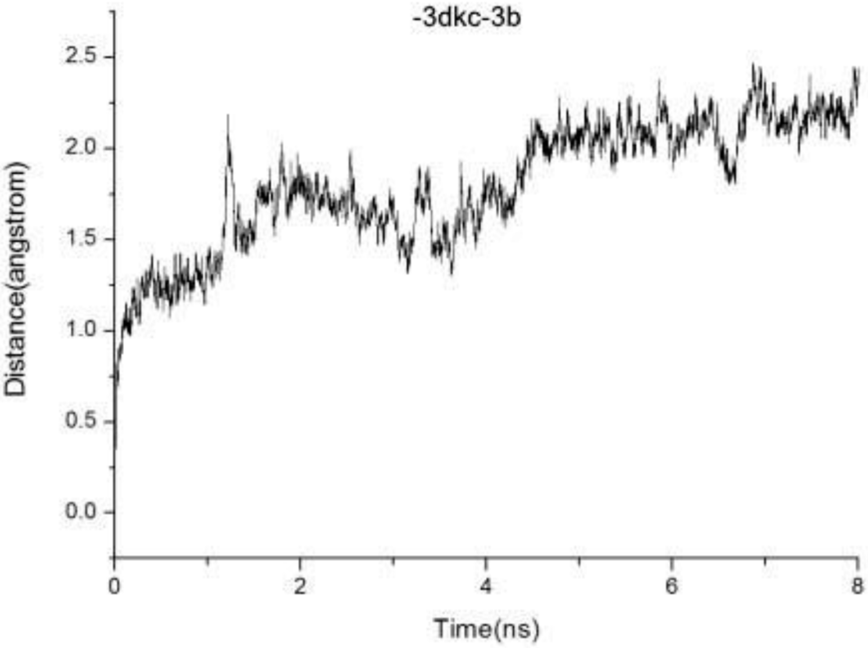

Supplementary Figure 4: Figures of Plots of RMSD for all of the backbone atoms (Å) vs simulation time (ns) for 3DKC in complex with 3b.

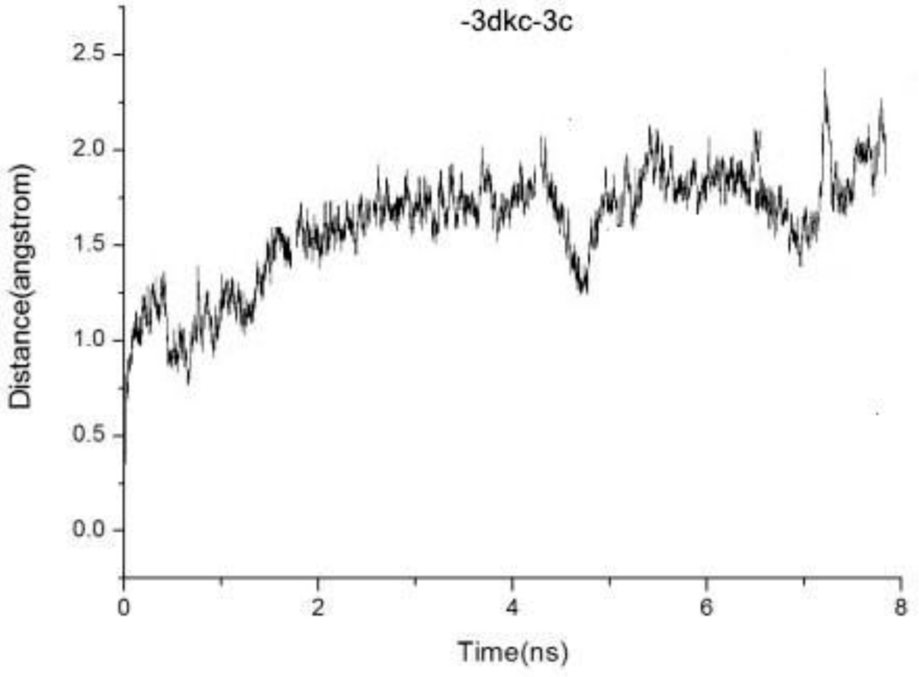

Supplementary Figure 5: Figures of Plots of RMSD for all of the backbone atoms (Å) vs simulation time (ns) for 3DKC in complex with 3c.

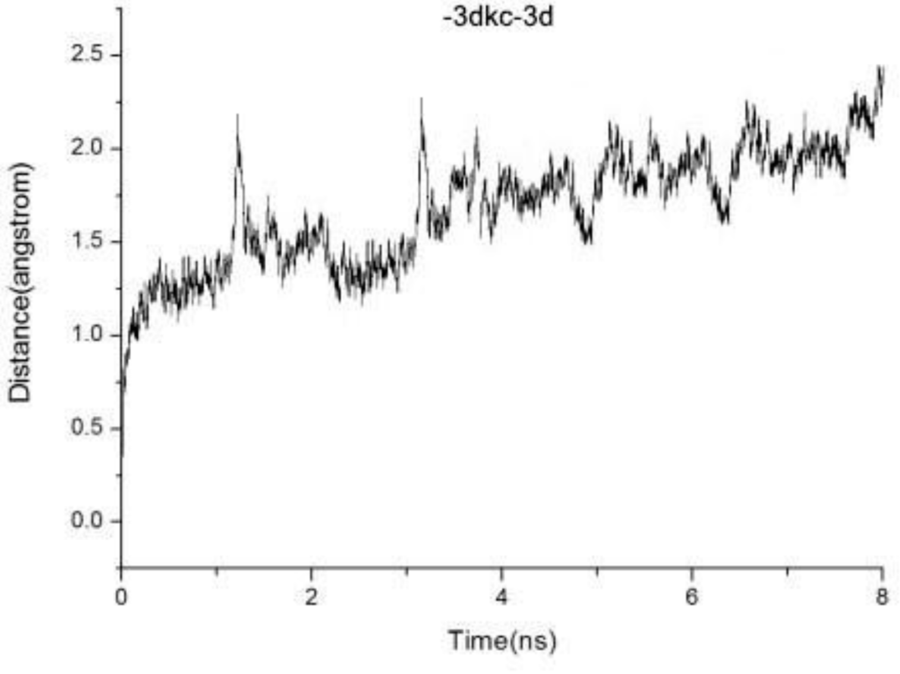

Supplementary Figure 6:Figures of Plots of RMSD for all of the backbone atoms (Å) vs simulation time (ns) for 3DKC in complex with 3d.

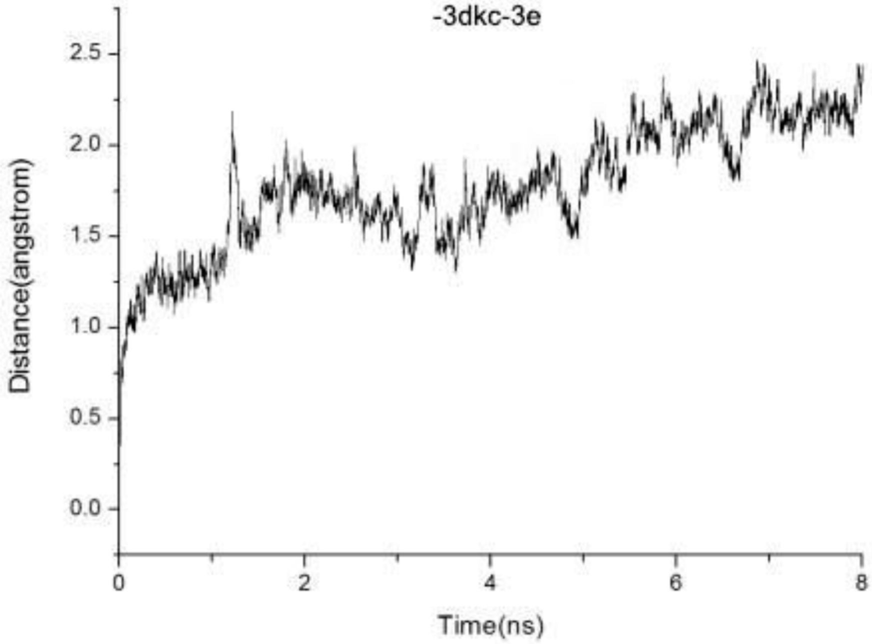

Supplementary Figure 7: Figures of Plots of RMSD for all of the backbone atoms (Å) vs simulation time (ns) for 3DKC in complex with 3e.

Supplementary Table 1: Inhibitory rate of compounds on c-Met kinase

Concentration (µM)	Inhibitory rate (%)						
	**1**	**2**	**3a**	**3b**	**3c**	**3d**	**3e**
0.19	23.44 ± 0.33	20.03 ± 0.27	26.33 ± 0.37	35.63 ± 0.44	33.77 ± 0.45	31.56 ± 0.27	28.28 ± 0.22
0.39	39.07 ± 0.45	34.93 ± 0.37	39.97 ± 0.43	46.91 ± 0.51	42.58 ± 0.51	40.88 ± 0.35	42.75 ± 0.35
0.78	46.98 ± 0.55	43.15 ± 0.52	47.22 ± 0.57	54.47 ± 0.47	47.99 ± 0.62	47.08 ± 0.44	48.01 ± 0.39
1.56	58.79 ± 0.61	55.22 ± 0.61	58.55 ± 0.69	63.38 ± 0.66	59.61 ± 0.66	59.03 ± 0.57	61.36 ± 0.64
3.12	65.38 ± 0.67	59.88 ± 0.53	64.98 ± 0.74	67.05 ± 0.59	64.54 ± 0.73	64.22 ± 0.59	67.55 ± 0.77
6.25	69.22 ± 0.77	64.16 ± 0.64	69.01 ± 0.76	72.56 ± 0.67	69.97 ± 0.81	68.98 ± 0.74	70.31 ± 0.59
12.50	73.69 ± 0.89	69.98 ± 0.59	74.58 ± 0.81	77.84 ± 0.81	73.79 ± 0.69	75.54 ± 0.88	75.69 ± 0.78
25.00	77.47 ± 0.97	74.21 ± 0.69	79.27 ± 0.85	81.24 ± 0.77	78.91 ± 0.87	80.61 ± 0.84	79.97 ± 0.91
50.00	82.09 ± 1.21	78.16 ± 0.88	84.26 ± 0.93	85.96 ± 0.93	83.69 ± 0.95	85.37 ± 0.96	85.33 ± 0.87
100.00	87.96 ± 1.01	81.18 ± 0.94	86.92 ± 0.97	88.78 ± 1.11	87.37 ± 1.20	87.74 ± 0.95	88.39 ± 0.99
